# Double Sequence Defibrillation for Out-of-hospital Cardiac Arrest: Unlikely Survival

**DOI:** 10.5811/cpcem.2018.7.38348

**Published:** 2018-08-15

**Authors:** Andrew Zabel, James R. Bence, Kyle Couperus

**Affiliations:** * William Beaumont Army Medical Center, El Paso, Texas; † Community Health Network, Department of Emergency Medical Services, Indianapolis, Indiana; ‡ Madigan Army Medical Center, Department of Emergency Medicine, Tacoma, Washington

## Abstract

Survival from out-of-hospital cardiac arrest (OHCA) is highest with early defibrillation and immediate, high-quality cardiopulmonary resuscitation. Return of spontaneous circulation (ROSC) is rare in OHCA. The purpose of this discussion and case report is to highlight the use of double sequence defibrillation (DSD) for refractory ventricular fibrillation (RVF). We present a 58-year-old male with RVF who successfully achieved ROSC after 38 minutes using DSD and had a good neurological outcome. DSD has shown promise in many case reports and case series as a means of increasing ROSC and survival rates in OHCA.

## INTRODUCTION

The global incidence of out-of-hospital cardiac arrest (OHCA) is 55 per 100,000 adults per year, and the average survival (to hospital discharge) is 7% in adults.^1^ Ventricular fibrillation (VF) is the most common rhythm after OHCA occurring in approximately 70% of cases.^2^ Return of spontaneous circulation (ROSC) is rare in OHCA; however, recently improved prehospital ROSC rates have been demonstrated.^3^ These moderately improved rates are likely attributed to high-quality bystander cardiopulmonary resuscitation (CPR) with increased emphasis on uninterrupted CPR with good technique.

We report on a case where double sequence defibrillation (DSD) was used to treat refractory ventricular fibrillation (RVF). RVF is typically defined as persistent VF following three to five unsuccessful shocks. DSD has been used safely in electrophysiology labs for quite some time.^4^ When using DSD, two sets of pads are placed in the anteroapical and anteroposterior positions and deliver a shock nearly simultaneously. (Image)

## CASE REPORT

A 58-year-old male with a history of hypertension and cardiac stents two years prior had just arrived at a Saturday morning prayer breakfast at church when he suddenly went unresponsive and was found to be pulseless and apneic by bystanders. CPR was immediately initiated. Emergency medical services (EMS) arrived with a six-minute response time. The patient was found to be in VF and defibrillated at 200 joules (J) unsuccessfully. Intraosseous access was established and epinephrine was administered. A supraglottic airway device was placed for ventilations and amiodarone 300mg was given. Over the course of 26 minutes five subsequent standard defibrillations were administered unsuccessfully.

At this point EMS contacted online medical control, and DSD was ordered. The patient had a second set of pads placed in the anteroapical position. Both the engine and medic defibrillators were used to deliver 360J, each nearly simultaneously. The post-shock rhythm revealed pulseless electrical activity (PEA) on the monitor, and CPR was resumed along with the seventh dose of epinephrine. The patient then was noted to be back in ventricular fibrillation, and dual sequential defibrillation was again performed. The post-shock rhythm analysis revealed a brief period of systole into sinus bradycardia with the ROSC. ROSC was achieved 38 minutes from the time of 911 dispatch.

En route to the hospital, the patient again lost pulses and was found to be in PEA. On arrival to the emergency department, he was confirmed pulseless, and CPR was continued. Point-of-care ultrasound showed cardiac activity shortly after ROSC was achieved again. The patient was intubated and central venous access established with norepinephrine infusion to stabilize blood pressure. His electrocardiogram revealed ST-segment elevation in augmented vector left (aVL) and the patient was urgently taken to the cardiac catheterization lab where he was found to have 100% occlusion of his circumflex artery and had a single stent placed. The patient was transferred to the intensive care unit where he was extubated less than 24 hours later. The patient was then discharged to home after only six days in the hospital. His inpatient echo showed an ejection fraction of 60%. At 30 days post-arrest he had some minor, short-term memory issues but had returned to work. The only complication was a lower extremity deep vein thrombosis.

## DISCUSSION

What makes this case remarkable is that this patient made a full neurological recovery despite the unlikelihood based on a recent publication out of Japan. Goto et al. showed that survival after 30 minutes of cardiac arrest was only 0.8% in a study group of over 17,000 cardiac arrest patients. Neurologically intact survival was only 0.4% in the same group after 30 minutes of cardiac arrest.^6^ This particular patient survived after 38 minutes with a cerebral performance category (CPC) score of one. The CPC score is a five-category scale for measuring neurological status after cardiac arrest (Table). A CPC score of one correlates to conscious and alert with good cerebral performance. A CPC score of five correlates to brain dead, circulation preserved.^7^ Many other factors most likely contributed to the good outcome of this patient’s case. First and foremost, this patient received immediate CPR after beginning cardiac arrest.

DSD is a tool that more physicians and EMS providers should become aware of. Several case reports and case series have been published in recent years supporting the potential use of DSD in OHCA. One case series by Cortez et al. showed a survival to discharge in three of 12 patients who presumably would have died if DSD had not been used after RVF. Two of the three patients (2/12, 17%) had a CPC score of one in the study.^8^ Merlin et al. showed even better results in another case series. three patients out of seven (3/7, 43%) in whom DSD was used after RVF survived to hospital discharge; once again these were patients who otherwise would presumably not have survived without the use of DSD. All three patients were discharged with CPC scores of one.^9^ Lastly, a less-successful case series by Cabanas et al. had 10 patients in whom DSD was used, but none survived to discharge. Three patients (3/10, 30%) achieved ROSC in the field with the utilization of DSD.^10^ An important discussion point is the presumed survival due to DSD; this caveat should not be ignored. Without further research we do not know if the next standard defibrillation attempt would have converted the patient from RVF.

**CPC-EM Capsule**

What do we already know about this clinical entity?

Return of spontaneous circulation (ROSC) is rare in out-of-hospital cardiac arrest (OHCA). Double sequence defibrillation (DSD) has shown promise as a means of increasing ROSC and survival rates in OHCA.

What makes this presentation of disease reportable?

Our patient achieved ROSC after 38 minutes of DSD, and survived neurologically intact. To our knowledge, this is the longest length of refractory ventricular fibrillation (RVF) time that was corrected using DSD.

What is the major learning point?

While effective and immediate cardiopulmonary resuscitation is most important for OHCA survival, other modalities should be considered for RVF including DSD.

How might this improve emergency medicine practice?

This case study helps justify further emergency medical services’ research investigating new standards of care for OHCA.

Current guidelines have established that survival from OHCA is highest with early defibrillation and immediate, high-quality CPR.^5^ We would like to suggest that early use of DSD defibrillation may contribute to survival from OHCA. In the above trials mentioned, the average time from the determination of arrest to first DSD was 32.3 minutes. In those patients who achieved ROSC, it was 31.3 minutes. In the patients who survived, average time to first DSD was 24.2 minutes. While these data suggest that earlier DSD could potentially improve outcomes, further research is needed. The patient we present achieved ROSC after 38 minutes of VF and survived neurologically intact.

## CONCLUSION

Further research is required to establish DSD as an effective means for treating RVF. DSD has shown promise in these case reports and case series as a possible tool of increasing ROSC and survival rates in OHCA. We present a case that adds to this literature and shows that positive outcomes can be achieved beyond the time window generally accepted if coupled with effective and immediate CPR.

Documented patient informed consent and/or Institutional Review Board approval has been obtained and filed for publication of this case report.

## Figures and Tables

**Image:**
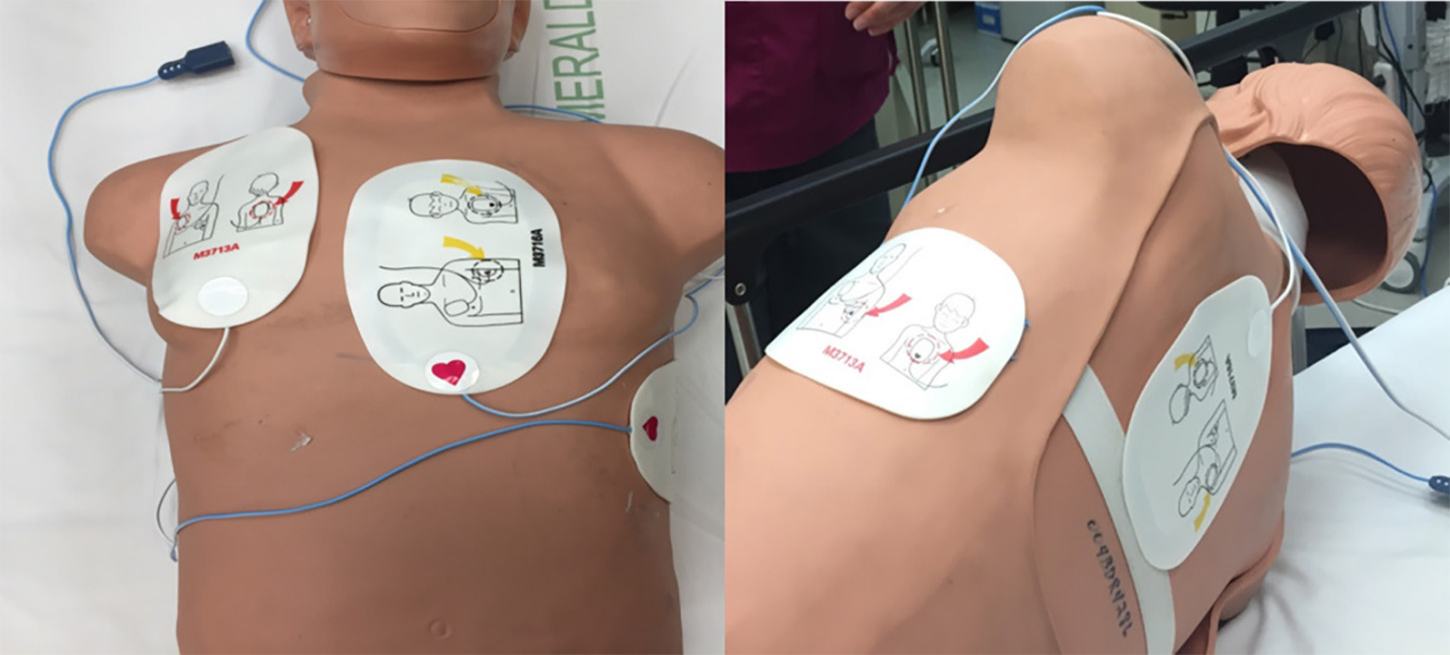
Pad placement for double sequence defibrillation, two sets of pads are placed in the anteroapical (red arrows) and anteroposterior (yellow arrows) positions as demonstrated on mannequin.

**Table:** Cerebral performance category (CPC) score: measuring neurological status after cardiac arrest.

CPC score	Definition
1	Conscious and alert with normal function or only slight disability
2	Conscious and alert with moderate disability
3	Conscious with severe disability
4	Comatose or persistent vegetative state
5	Brain dead or death from other causes

## References

[1] Berdowski J, Berg RA, Tijssen JGP (2010). Global incidences of out-of-hospital cardiac arrest and survival rates: Systematic review of 67 prospective studies. Resuscitation.

[2] Leacock BW (2014). Double simultaneous defibrillators for refractory ventricular fibrillation. J Emerg Med.

[3] Daya MR, Schmicker RH, Zive DM (2015). Out-of-hospital cardiac arrest survival improving over time: Results from the Resuscitation Outcomes Consortium (ROC). Resuscitation.

[4] Hoch DH, Batsford WP, Greenberg SM (1994). Double sequential external shocks for refractory ventricular fibrillation. J Am Coll Cardiol.

[5] Nolan JP, Hazinski MF, Aickin R (2015). Part 1: Executive summary: 2015 International Consensus on Cardiopulmonary Resuscitation and Emergency Cardiovascular Care Science with Treatment Recommendations. Resuscitation.

[6] Goto Y, Maeda T, Nakatsu-Goto Y (2013). Neurological outcomes in patients transported to hospital without a prehospital return of spontaneous circulation after cardiac arrest. Crit Care.

[7] Rittenberger JC, Raina K, Holm MB (2011). Association between cerebral performance category, modified Rankin scale, and discharge disposition after cardiac arrest. Resuscitation.

[8] Cortez E, Krebs W, Davis J (2016). Use of double sequential external defibrillation for refractory ventricular fibrillation during out-of-hospital cardiac arrest. Resuscitation.

[9] Merlin MA, Tagore A, Bauter R (2016). A case series of double sequence defibrillation. Prehosp Emerg Care.

[10] Cabanas JG, Myers JB, Williams JG (2015). Double sequential external defibrillation in out-of-hospital refractory Ventricular fibrillation: a report of ten cases. Prehosp Emerg Care.

